# Differentially Expressed MicroRNAs in the Development of Early Diabetic Retinopathy

**DOI:** 10.1155/2017/4727942

**Published:** 2017-06-15

**Authors:** Qiaoyun Gong, Jia'nan Xie, Yang Liu, Ying Li, Guanfang Su

**Affiliations:** Eye Center, The Second Hospital of Jilin University, No. 218 Ziqiang Street, Changchun, Jilin 130021, China

## Abstract

The pathological mechanisms of diabetic retinopathy (DR), a leading cause of blindness in adults with diabetes mellitus, remain incompletely understood. Because microRNAs (miRNAs) represent effective DR therapeutic targets, we identified aberrantly expressed miRNAs associated with cellular dysfunction in early DR and detected their potential targets. We exposed human retinal endothelial cells (HRECs) and a cell line of retinal pigment epithelial (RPE) cells to high glucose (25 mmol/L, 1–7 days) to mimic DR progression and used streptozotocin-injected rats (4–8 weeks) for an in vivo diabetes model. HREC/RPE viability decreased after 24 h incubation and diminished further over 6 days, and Hoechst staining revealed hyperglycemia-induced HREC/RPE apoptosis. Although miR-124/-125b expression decreased with DR progression in vitro and in vivo, miR-135b/-199a levels decreased in retinal cells under hyperglycemia exposure, but increased in diabetic retinas. Moreover, miR-145/-146a expression decreased gradually in high-glucose-treated HRECs, but increased in hyperglycemia-exposed RPE cells and in diabetic rats. Our findings suggested that aberrant miRNA expression could be involved in hyperglycemia-induced retinal-cell dysfunction, and the identified miRNAs might vary in different retinal layers, with expression changes associated with DR development. Therefore, miRNA modulation and the targeting of miRNA effects on transcription factors could represent novel and effective DR-treatment strategies.

## 1. Introduction

Diabetic retinopathy (DR) is currently the main cause of visual disability and blindness in working-age adults (20–65 years old) [1, 2]. DR results from abnormal retinal blood vessels that are either nonproliferative or proliferative. The blood-retinal barrier (BRB), which comprises the retinal vasculature and the retinal pigment epithelium, excludes the neural elements of the retina and the cytotoxic products from circulating inflammatory cells, thereby protecting and allowing the retina to regulate its extracellular chemical composition [3]. The inner BRB formed by retinal capillaries features endothelial cells harboring tight junctions that are almost impermeable to protein transport [4]. Endothelial cells are responsible for maintaining the BRB, and their impairment causes increased vascular permeability [5]. The outer BRB, which is formed by retinal pigment epithelial (RPE) cells, is located between retinal photoreceptors and the choroid. RPE cells play a crucial role in fluid balance within the retina [6] by forming the outer BRB and supporting the function of photoreceptors, with RPE cells capable of activation via milieu changes [7]. In the pathogenesis of diabetic complications, the primary causal factor is considered to be lasting exposure to hyperglycemia [8]. Exposure to high glucose (HG) and the resultant damage are pivotal to the BRB imbalance that leads to the leakage of fluids and lipids into the retina and contributes to DR progression. Therefore, to investigate the molecular changes induced by HG and assess the effects of glucose on the retina, we conducted in vitro studies using human retinal endothelial cells (HRECs) and RPE cells cultured under hyperglycemia conditions for different periods. Furthermore, for in vivo studies, we established a rat model of diabetes mellitus (DM) by intraperitoneally injecting the animals with streptozotocin (STZ).

DR pathogenesis is a multifactorial process, and numerous protein-encoding genes related to hyperglycemia-linked pathways have been investigated in DR progression [1, 9]. However, emerging data also suggest that an enormous number of noncoding RNAs, which exhibit little or no protein-coding potential, are expressed and play critical roles in DR pathogenesis [10–12], with microRNAs (miRNAs), in particular, receiving considerable research attention. miRNAs are a group of short (~21–23 nucleotides), highly conserved endogenous RNAs that do not encode proteins. miRNAs modulate gene expression through transcriptional or posttranscriptional regulation, inducing mRNA degradation or inhibiting protein translation by binding to the seed region in the 3′-untranslated region (3′-UTR) of target genes [13–15]. Notably, miRNAs are involved in the proliferation, migration, and apoptosis of retinal cells and in DR-related neovascularization, and miRNAs that exhibit increased or decreased expression during DR pathogenesis have been identified [10]. Modulation of miRNA levels exerts beneficial effects on slowing DR progression and could potentially be employed in DR-therapeutic strategies. However, few studies have focused on or elucidated the progressive changes in miRNA levels in DR. Wu et al. [11] reported that the expression of certain miRNAs either increases (e.g., miR-182) or decreases (e.g., miR-10b) in parallel with DR progression in the retinas of rats with STZ-induced DM; however, no study has been conducted thus far to investigate miRNA variations in human retinal cells under prolonged HG exposure. To effectively use miRNAs in clinical applications for DR, it is crucial to identify the continuous alterations of miRNAs related to DR development and pathogenesis. Here, based on the results of bioinformatics analysis, we examined the expression of hsa-miR-124-3p, -125b-5p, -135b-5p, -145-5p, -146a-5p, and -199a-5p in HRECs or RPE cells under hyperglycemic conditions or in vivo in diabetic rats.

In this study, we also focused on specificity protein 1 (SP1), which could represent a previously unknown target of miR-125b-5p. SP1 belongs to a family of transcription factors that harbors GC-rich promoters and is involved in basal promoter activity. SP1 positively regulates the expression of diverse genes encoding various angiogenesis-related factors, including vascular endothelial growth factor (VEGF) and fibrogenic cytokine, which are related to cell proliferation and migration, BRB damage and neovascularization, and, eventually, visual impairment. These factors can be detected in the vitreous fluid collected from patients with proliferative DR [16]. Therefore, combined miRNA and SP1 targeting to manipulate the pathways regulated by the transcription factor might represent a favorable strategy in the treatment of DR.

Our objectives were to discover the detailed alterations of additional miRNAs associated with the dysfunction and apoptosis of retinal cells in the early stage of DR. Furthermore, we analyzed the potential of therapeutic strategies based on targeting miRNA effects on transcription factors in the DR process. In addition to revealing the roles of miRNAs in DR, our study provides insights into miRNA expression in chronic diseases and potentially helps improve the development of therapeutic strategies used for these diseases.

## 2. Materials and Methods

### 2.1. Cell Culture

HRECs were purchased from ANGIO-PRO TEOMIE (Boston, MA, USA) and maintained in endothelial cell medium containing 5% fetal bovine serum (FBS) and 1% endothelial cell growth supplement (ScienCell, Carlsbad, CA, USA). The human RPE cell line ARPE-19 was purchased from American Type Culture Collection (ATCC, Manassas, VA, USA) and cultured according to the manufacturer's instructions. The cells were maintained in Dulbecco's modified Eagle medium/F-12 (Hyclone, Beijing, China) containing 10% FBS (Gibco; Thermo Fisher Scientific, Waltham, MA, USA). HREC and ARPE-19 cultures were maintained at 37°C in a humidified atmosphere containing 5% CO_2_.

### 2.2. Hyperglycemia Treatment

HRECs and ARPE-19 cells were plated at 2500 cells/cm^2^ in 6-well plates (Corning; Acton, MA, USA) and treated with normal glucose (NG; 5.5 mmol/L) as a control or with HG (25 mmol/L) or the osmotic control mannitol (MN; 19.5 mmol/L MN together with NG) under normoxic conditions for 1, 3, 5, and 7 days to mimic the chronic disease course in the early stage of DR. The cells were cultured in NG first and then in HG or in MN according to the number of HG days. The media were changed daily to eliminate metabolic byproducts and to provide the nutrients necessary for the cells.

### 2.3. Animal Model

Animal experiments were conducted according to the guidelines specified by the Animal Care and Use Committee of Jilin University. Male Sprague-Dawley rats (~200 g; 8 weeks old) were obtained from the Animal Center, College of Basic Medical Sciences, Jilin University, and housed in standard plastic rodent cages under a controlled environment (24°C; 12/12 h light/dark cycle). Diabetes was induced by injecting the animals once intraperitoneally with STZ [65 mg/kg, in citrate buffer (pH 4.5)]. Control rats received an identical volume of citrate buffer. Rats were regarded as diabetic when their blood glucose level exceeded 16.7 mmol/L at 72 h and 1 week post-STZ administration. Body weights of the rats were also monitored throughout the study. Control and diabetic rats were maintained for 4, 6, and 8 weeks (*n* = 6/group).

### 2.4. Cell Viability Assay

Cell viability was assessed using 3-(4,5-dimethylthiazol-2-yl)-5-(3-carboxy-methoxyphenyl)-2-(4-sulfophenyl)-2H–tetrazolium (MTS; Promega, Shanghai, China) according to the manufacturer's protocol. HRECs or ARPE-19 cells were seeded at a density of 2 × 10^3^ cells/well in 96-well plates and treated with NG or HG for 1, 2, 3, 4, 5, and 6 days. Before detection, 100 *μ*L of medium was replaced with fresh medium and 20 *μ*L of MTS was added to cells. Following incubation for 1.5 h at 37°C, the absorbance at 490 nm was measured.

### 2.5. Assessment of Hyperglycemia-Induced Apoptosis

To distinguish the type of cell death under hyperglycemia conditions, nuclear morphology was examined by means of Hoechst 33258 staining and fluorescence microscopy. HRECs and ARPE-19 cells were treated with HG for 1, 3, 5, and 7 days, washed twice with 1× phosphate-buffered saline, incubated with 0.1 *μ*g/mL Hoechst 33258 (Sigma-Aldrich, St. Louis, MO, USA) for 15 min in the dark, and examined under a microscope (Olympus, Tokyo, Japan). Cells harboring fragmented or condensed pyknotic nuclei were counted as apoptotic cells, and data are presented here as apoptotic cell percentages (apoptotic cells/total cells × 100).

### 2.6. miRNA Mimics and Transfection

The hsa-miR-125b-5p mimic and a scrambled negative-control miRNA were chemically synthesized by GenePharma (Shanghai, China). The sequences were, respectively, 5′-UCCCUGAGACCCUAACUUGUGA-3′ and 5′- UUCUCCGAACGUGUCACGUTT-3′. ARPE-19 cells in the logarithmic growth phase were seeded in 6-well plates and cultured under HG for 2 days before transfection. Upon reaching ~70% to 80% confluence, cells were transfected with miR-125b mimic or scramble-control miRNA (both at 50 nmol/L) using Lipofectamine RNAiMAX (Invitrogen, Carlsbad, CA, USA) transfection reagent. At 5 h after transfection, the medium was changed to fresh HG medium, and at 48 h and 72 h after transfection, the cells were harvested for mRNA and protein analysis, respectively.

### 2.7. Total RNA Isolation and Identification

Total RNA was extracted from HRECs and ARPE-19 cells cultured under different conditions or from rat retinal tissues. An Eastep Super total RNA extraction kit (Promega) was used according to manufacturer protocol, and RNA concentration and purity were measured using a NanoDrop 2000c spectrophotometer (Thermo Fisher Scientific). RNA samples featuring an A260/A280 value of ~2.0 were generally used for further analysis. The integrity of RNA samples was assessed through 1% agarose-gel electrophoresis.

### 2.8. Determination of miRNA- or mRNA-Expression Levels

To analyze miRNA-expression levels, 1.5 *μ*g of total RNA was polyadenylated and reverse transcribed using an All-in-One miRNA first-strand cDNA synthesis kit (GeneCopoeia, Rockville, MD, USA). The obtained cDNA was diluted 1 : 5 for further quantitative real-time polymerase chain reaction (PCR). miRNA levels were measured using an All-in-One miRNA quantitative PCR (qPCR) detection kit with specific primers (GeneCopoeia). Measurements were performed in triplicate on a LightCycler 480 (Roche Diagnostics, Basel, Switzerland), and RNA U6 was used as a normalization control for miRNA expression.

For mRNA analysis, 500 ng of total RNA was reverse transcribed using a Perfect real-time RT reagent kit (Takara Bio, Beijing, China) in a 20 *μ*L reaction volume, after which qPCR was performed using a reaction mixture that contained 1 *μ*L of the cDNA, 10 *μ*mol/L gene-specific primers (forward and reverse, mixed together), and 10 *μ*L of 2× Fast SYBR Green master mix (Roche Diagnostics). Three replicates for each biological mixture were used, and reactions were performed on a LightCycler 480 (Roche Diagnostics). For each miRNA or mRNA run, we included two negative controls containing water instead of the template. The relative expression levels of miRNAs and mRNAs were calculated using the 2^−ΔΔCt^ method, in which the ratio of expression between an experimental group and the control group was determined.

### 2.9. Western Blot

Total protein was collected from ARPE-19 cells by lysing the cells for 30 min on ice in a cell-lysis buffer [radioimmunoprecipitation assay buffer containing phenylmethylsulfonyl fluoride (Dingguo Changsheng, Beijing, China)], treating the lysates with ultrasound and then centrifuging the lysates at 12,000 rpm for 10 min at 4°C. Protein concentrations were determined using a bicinchoninic acid protein assay kit (Beyotime, Jiangsu, China). Supernatant proteins were collected, denatured, concentrated in 5% sodium dodecyl sulfate-polyacrylamide gel electrophoresis (SDS-PAGE) stacking gels, separated in 10% SDS-PAGE gels, and transferred to polyvinylidene difluoride membranes. The membranes were blocked in 5% skim milk for 1 h, incubated with primary antibodies against SP1 (1 : 150; Santa Cruz Biotechnology, Dallas, TX, USA) and *β*-actin (1 : 1000; CMC-TAG, Milwaukee, WI, USA) at 4°C overnight, and then incubated with secondary antibodies (1 : 5000; Boster, Wuhan, China) for 40 min. An enhanced chemiluminescence plus kit (Millipore, Billerica, MA, USA) was used to visualize stained bands, and the densities of the grey bands were determined using ImageJ software (National Institutes of Health, Bethesda, MD, USA).

### 2.10. Statistical Analysis

Each experiment was repeated at least three times. Experimental data are presented as means ± standard deviation. Analyses for statistical significance were performed using Prism 6.0 software (GraphPad Software, San Diego, CA, USA). To assess statistical differences between groups in multiple comparisons, we used one-way analysis of variance, followed by the Student-Newman-Keuls post hoc test. The presented *P* values are two-sided, and results were considered statistically significant at a *P* < 0.05.

## 3. Results

### 3.1. Effect of Hyperglycemia on HREC and ARPE-19 Cell Viability and Apoptosis

Our aim was to identify miRNAs exhibiting altered expression during the progression of early DR. Therefore, we conducted experiments on HRECs and ARPE-19 cells exposed to hyperglycemia (25 mmol/L glucose).

**Figure 1:**
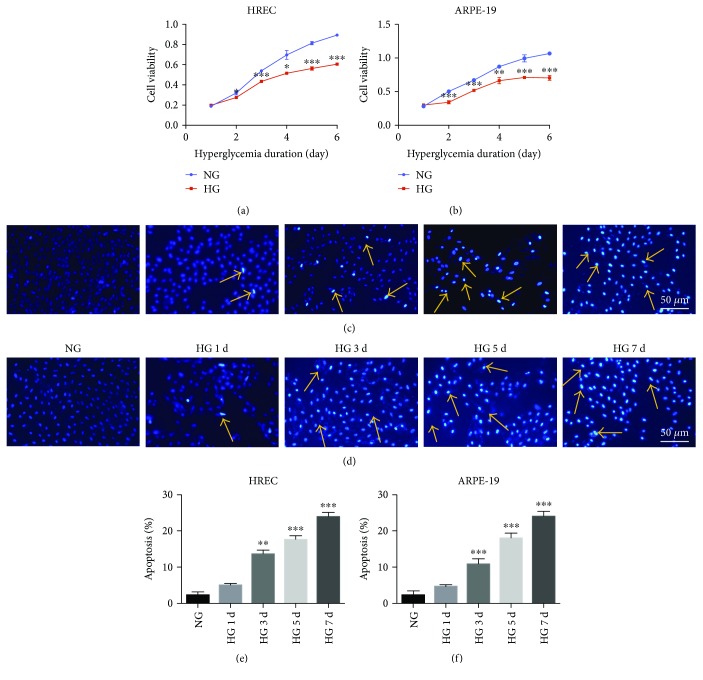
Effects of high glucose (HG) on the viability and apoptosis of HRECs and ARPE-19 cells. (a, b) Time-dependent increase in hyperglycemia-induced inhibition of HRECs and RPE proliferation. (c, d) Representative images of HRECs and RPE cells treated with normal glucose (NG) or HG. The number of apoptotic cells harboring condensed or fragmented nuclei (yellow, *arrowheads*) increased along with an increase in HG-treatment time. (e, f) Statistical analysis of apoptotic HRECs and RPE cells under different conditions. Quantification was performed using ImageJ software. ^∗^*P* < 0.05; ^∗∗^*P* < 0.01; ^∗∗∗^*P* < 0.001.

MTS assay results showed that with an increase in the time of hyperglycemia treatment up to 6 days, the viability of HRECs and RPE cells decreased significantly (Figures 1(a) and 1(b)). We also examined how HG exposure altered the phenotype of these cells. Hoechst DNA-dye nuclear staining indicated that hyperglycemia induced nuclear condensation or fragmentation, particularly after incubation for 5 and 7 days. Representative apoptotic HRECs and RPE cells harboring condensed nuclei are indicated by arrowheads in Figures 1(c) and 1(d). The results of statistical analysis confirmed the observed increase in apoptosis of HRECs and RPE cells under HG exposure (Figures 1(e) and 1(f)).

### 3.2. Hyperglycemia-Induced Changes in miRNA Expression in HRECs and RPE Cells

**Figure 2:**
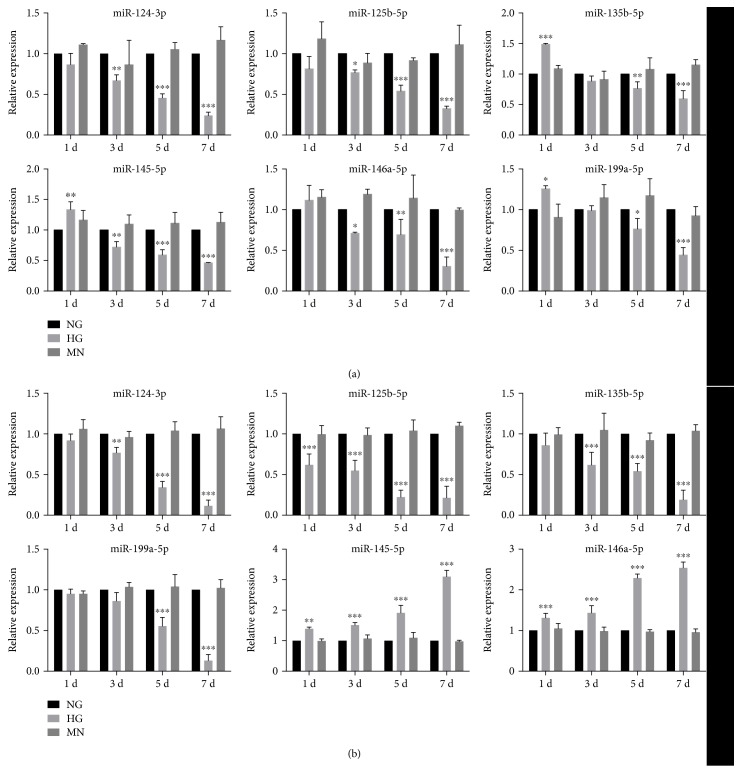
Aberrant levels of miRNAs in HRECs and ARPE-19 cells under hyperglycemia exposure for different periods. (a) In HRECs, miR-124-3p and -125b-5p were downregulated along with an increase in HG-exposure time, whereas miR-135b-5p, -145-5p, -146a-5p, and -199a-5p were first upregulated and then downregulated as compared to levels observed following NG treatment. (b) In RPE cells, HG treatment caused a gradual decline in the levels of miR-124-3p, -125b-5p, -135b-5p, and -199a-5p relative to levels observed after NG exposure. By contrast, miR-145-5p and -146a-5p levels were continually upregulated in RPE cells upon HG exposure. Treatment of HRECs and RPE cells with mannitol (MN) revealed that osmotic change exerted no effect on miRNA expression. ^∗^*P* < 0.05; ^∗∗^*P* < 0.01; ^∗∗∗^*P* < 0.001.

To investigate the relevance of the miRNAs exhibiting expression changes with DR progression, we measured miRNA levels in HRECs cultured under HG exposure for different periods. Real-time qPCR analysis revealed that the levels of several miRNAs changed during the early stage of DR. Although the expression of miR-124-3p and -125b-5p decreased gradually with an increase in HG-exposure time and concomitant with the early development of DR, miR-135b-5p, -145-5p, -146a-5p, and -199a-5p were upregulated on the first day and then downregulated on the following days (Figure 2(a)). These changes indicated that over the course of further DR development, the identified miRNAs might perform distinct (perhaps even opposite) functions. Our results suggested that miRNA levels likely vary during DR progression and that the roles played by miRNAs in retinal endothelial cells are complex and warrant further investigation.

We then exposed ARPE-19 cells to HG for different periods in an identical manner as HRECs to mimic the course of DR. The results of qPCR analysis showed that some of the miRNAs identified here were expressed in a manner distinct from that in HRECs. The expression of miR-124-3p, -125b-5p, -135b-5p, and -199a-5p decreased gradually along with an increase in HG-exposure time, whereas the expression of miR-145-5p and -146a-5p was elevated from day 1 to 7 (Figure 2(b)), with the levels of these two miRNAs on day 7 > 2.5-fold higher than those in the NG-exposed group. Moreover, in the MN osmotic-control group, no notable changes were detected in any of the miRNAs analyzed. Therefore, our results suggested that the levels of aberrantly expressed miRNAs varied in retinal cells concomitantly with DR development. These findings indicated that at different stages of DR, miRNA-based therapy should be based on the miRNAs that can be modulated functionally and markedly.

### 3.3. Aberrantly Expressed miRNAs in the Retinas of Diabetic Rats

**Figure 3:**
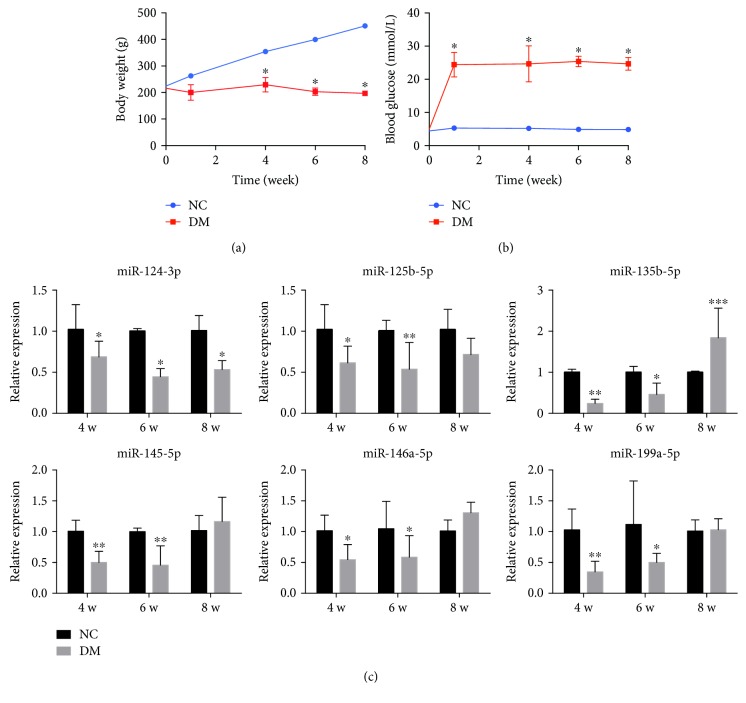
Abnormal expression of miRNAs in the retinas of rats with STZ-induced diabetes. (a) Body weight and (b) blood glucose levels of normal control (NC) and DM rats (*n* = 6/group). After intraperitoneal injection of STZ (65 mg/kg) in citrate buffer (pH 4.5), the body weight of DM rats was significantly lower and blood glucose levels increased 5-fold relative to the corresponding values measured for NC rats. (c) Expression of miR-124-3p and -125b-5p was downregulated in parallel with DR progression, whereas the levels of miR-135b-5p, -145-5p, -146a-5p, and -199a-5p decreased during the early stage of DR before increasing in the later stages of DR. ^∗^*P* < 0.05; ^∗∗^*P* < 0.01; ^∗∗∗^*P* < 0.001, compared with NC.

To examine abnormal expression of miRNAs in the DM retina, we established an animal model of DM by injecting rats with STZ. These DM rats exhibited significant weight loss as compared with age-matched control rats (Figure 3(a)), with weight loss detected at 1 week post-STZ injection remaining low over the course of DR progression. Moreover, blood glucose levels were markedly elevated in STZ-treated rats during the first week after injection (Figure 3(b)), with levels >5-fold higher than those in normal control rats.

We then used retinal tissues from diabetic and control rats for miRNA validation through real-time PCR analysis. Unexpectedly, the expression of only miR-124-3p and -125b-5p was lower in the retina of DM rats in relation to DR development. By contrast, miR-135b-5p, -145-5p, -146a-5p, and -199a-5p levels were lower at 4 and 6 weeks post-STZ injection before increasing after 8 weeks to levels exceeding even those of the controls (Figure 3(c)). These results and those presented in the preceding subsection together showed that both in vitro and in vivo, the expression of miR-124 and -125b decreased in correspondence with DR progression, but that of miR-135b and -199a decreased in retinal cells under hyperglycemia exposure and increased in the DM retina. Moreover, miR-145 and -146a were downregulated gradually in HG-treated HRECs, but were upregulated in RPE cells exposed to hyperglycemia and in DM rats.

### 3.4. The Role of SP1, a Previously Unknown Potential Target of miR-125b-5p, in DR Progression

Previous work revealed miR-125b-5p as one of the most abundant miRNAs in the retina, with this miRNA exhibiting an inherent ability to regulate cell differentiation, growth, and development [17, 18]. In this study, we confirmed that miR-125b was expressed at low levels in both HRECs and ARPE-19 cells under hyperglycemia exposure and in the DM retina. Therefore, modulation of miR-125b in early DR might exert beneficial effects with regard to slowing DR progression and DR-associated impairment. Furthermore, it is critical to study miR-125b-target genes related to the DR pathogenesis; however, only a few mRNAs have thus far been verified as miR-125b targets involved in DR pathogenesis [17]. According to the prediction obtained using TargertScan (http://www.targetscan.org/vert_71/), we identified SP1 as a target of miR-125b-5p; therefore, to examine potential regulatory interactions between miR-125b and SP1 during the course of DR, we used qPCR to measure SP1 mRNA levels in HG-treated HRECs and RPE cells. Subsequently, we transfected an miR-125b-5p mimic into ARPE-19 cells under hyperglycemia exposure, confirmed miR-125b upregulation, and determined the SP1-expression level at both the mRNA and protein levels.

**Figure 4:**
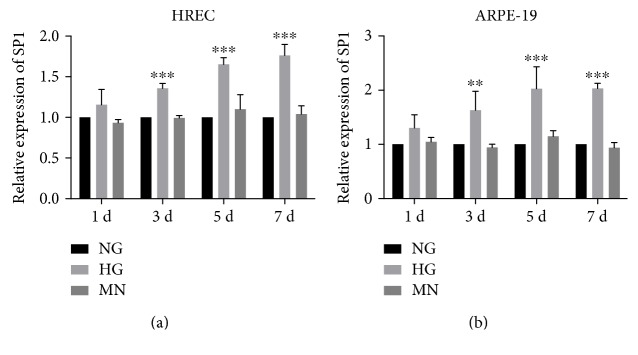
Quantitative reverse transcription PCR analysis showing SP1 mRNA upregulation in both HRECs and RPE cells exposed to hyperglycemia. SP1 expression in (a) HRECs and (b) RPE cells increased with HG-exposure time. SP1 mRNA levels were normalized relative to the mRNA levels of PUM1 and PPIA in HRECs and RPE cells, respectively. Culturing of HRECs and RPE cells with MN revealed that osmotic change did not affect SP1 expression. ^∗∗^*P* < 0.01; ^∗∗∗^*P* < 0.001, compared with NG.

**Figure 5:**
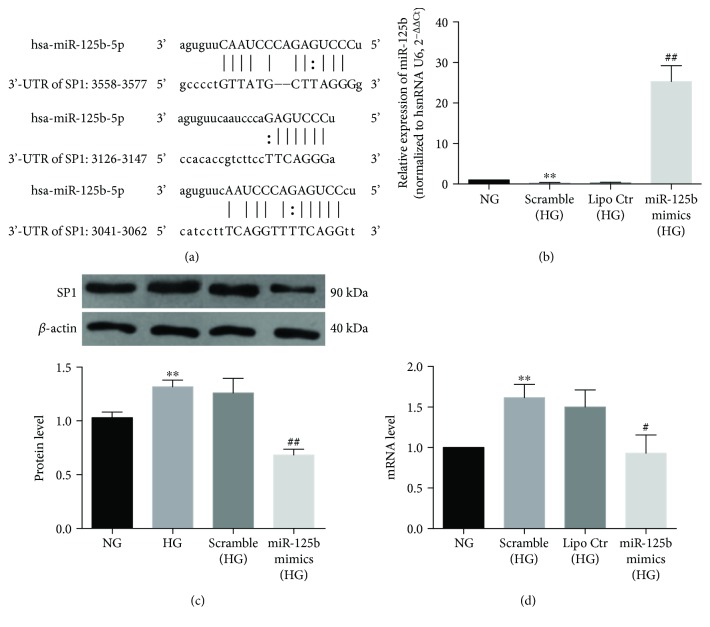
SP1 might represent a previously unknown target of miR-125b and suppression of high levels of SP1 expression by miR-125b in RPE cells under hyperglycemia. (a) Three representative predicted seed regions of miR-125b in the 3′ UTR of SP1 mRNA. (b) Expression of miR-125b was significantly increased after transfection with an miR-125b mimic (as compared to levels after transfection with a scramble-control miRNA) in RPE cells cultured under HG exposure. Lipofectamine did not affect miR-125b expression. (c, d) Hyperglycemia-induced increases in SP1 levels in RPE cells were alleviated by miR-125b upregulations, which was confirmed by (c) western blot and (d) quantitative reverse transcription PCR. Endogenous *β*-actin was used as a reference for protein expression, and SP1 mRNA level was normalized relative to PPIA mRNA level. ^∗∗^*P* < 0.01, compared with NG; ^#^*P* < 0.05; ^##^*P* < 0.01, compared with scramble control.

Over increasing durations of hyperglycemia exposure, SP1 mRNA levels were evaluated in HRECs (Figure 4(a)) and ARPE-19 cells (Figure 4(b)) after normalization relative to the mRNA levels of Pumilio homolog 1 (PUM1) and peptidylprolyl isomerase A (PPIA), respectively. PUM1 in HRECs and PPIA in ARPE-19 cells were selected for normalization based on our previous analysis [19, 20]. In the MN osmotic-control groups, SP1 levels did not change significantly; however, when HRECs and ARPE-19 cells were exposed to HG, SP1 levels increased and remained high until days 5 and 7. This increase in SP1 expression along with DR progression was the opposite of that observed with miR-125b levels. As shown in Figure 5(a), putative interaction sites for miR-125b are present in the 3′-UTR of SP1 mRNA; therefore, we directly tested the effect of an miR-125b mimic on SP1 levels. Transfection of the miR-125b mimic led to a 25-fold increase in the level of miR-125b relative to a level in cells transfected with scramble-control miRNA (Figure 5(b)). Notably, after introduction of miR-125b into cells, SP1 expression was significantly inhibited at both the protein and mRNA levels (Figures 5(c) and 5(d)). These results strongly suggested that SP1 is a previously unidentified direct target of miR-125b associated with the progression of DR. Therefore, we propose that miR-125b could restrict SP1 levels in cells and that this modulation might affect its downstream targets and thereby ameliorate DR progression. These possibilities will be further and specifically tested in future studies.

## 4. Discussion

An uncontrolled high level of blood glucose is the leading cause of type 2 DM and related complications. DR is a chronic and serious eye complication associated with DM, and people with DR present an increased risk of other microvascular and macrovascular complications associated with diabetes [21]. Hyperglycemia is closely related to the pathologic changes related to DR, which are responsible for induction of neovascularization, inflammation, oxidative stress, apoptosis, and cell proliferation [22, 23]. In the early stages of DR, HRECs and RPE cells, which are components of the BRB, are affected and impaired by the adverse effects of HG, subsequently inducing BRB dysfunction and contributing to DR progression. Retinal-cell dysfunction is characterized by a decrease in cell viability and increase in apoptosis [24]. In this study, HRECs and RPE cells exposed to 25 mmol/L glucose for various periods exhibited diminished cell viability and increased apoptosis. These observed changes agreed with the findings reported in the previous studies.

Noncoding RNAs have been investigated in diverse diseases, including neuronal diseases, cardiovascular diseases, and cancer, with miRNAs, in particular, playing key roles in these diseases. Increasing numbers of miRNAs aberrantly expressed during DR progression have been identified, with some even applied to clinical therapies [10] or, in the case of circulating miRNAs, used as DR biomarkers [25]. To identify the potential role played by miRNAs in glucose-induced retinal-cell dysfunction/apoptosis, we analyzed miRNA expression related to DR by conducting in vitro studies on cells exposed to hyperglycemia and in vivo studies on a rat model of type 2 DM. Initial results obtained from the in vitro study (on HG-treated HRECs and ARPE-19 cells) showed that miRNAs were markedly dysregulated under hyperglycemic conditions. Among the identified miRNAs, miR-124-3p, -125b-5p, -135b-5p, and -199a-5p showed a gradual decrease in expression in either HRECs or RPE cells along with an increase in HG-treatment time, whereas miR-145-5p and -146a-5p exhibited opposite expression changes (downregulation in HRECs and upregulation in RPE cells) in response to HG exposure for increasing durations. Moreover, the same miRNA could be distinctly expressed in the different layers of the retina under the same conditions. Independent analysis of the miRNA data from the in vivo study on diabetic rats revealed changes in miRNA expression associated with DR progression. Although miR-124-3p and -125b-5p remained downregulated during the different periods of DR examined, miR-135b-5p, -145-5p, -146a-5p, and -199a-5p exhibited increases in expression along with DR development. These four miRNAs were expressed at lower levels than those observed in control rats at 4 and 6 weeks post-STZ induction, followed by an increase to levels exceeding those of controls at week 8. These findings implied that the same miRNAs might play opposite roles during DR development, a possibility also supported by previous studies. In a rat model of STZ-induced diabetes, miR-146a was downregulated in retinal endothelial cells at 1 month after diabetes onset [26], but was upregulated in the retina and in retinal endothelial cells at 3 months after diabetes onset [27], which agreed with our findings. The alteration of miRNAs in early stages of DR has been observed not only in human retinal cells, but also in the retinas of diabetic rats. The underlying reason for these findings could be that the pathologic changes associated with DR progression exert distinct effects on miRNA biogenesis, resulting in either upregulation or downregulation at different stages of DR. Therefore, the application of miRNAs in clinical therapy should be based on the modulation of miRNAs for which stable and confirmed changes in expression have been reported.

Among the miRNAs studied here, miR-124-3p and -125b-5p were found to be downregulated during DR progression both in vitro and in vivo. MiR-124, which was identified as a tumor suppressor, has been confirmed to be downregulated in cancers and investigated for its role in regulating cell proliferation and apoptosis by targeting different genes in distinct types of cancer [28–30]. Moreover, miR-135b was identified as a biomarker for colorectal cancer [31] and reported to modulate apoptosis by targeting large tumor-suppressor kinase 2 [32]. However, the role of these miRNAs in glucose-induced retinal-cell dysfunction during DR progression has not been studied previously. MiR-145 was shown to participate in the insulin-resistance pathway involving AKT/protein kinase B, which is strongly associated with type 2 DM [33]. The potential functions of miR-124, -135b, and -145 warrant further investigation regarding their roles in DR pathogenesis. Previous work showed that miR-146a was upregulated and functioned in negative-feedback regulation of the nuclear factor (NF)-*κ*B-activation pathway in the retinal endothelial cells of diabetic rats at 3 months after diabetes onset [27]. The negative-feedback effect on NF-*κ*B activation suggests that miR-146a could serve as an alternative target for DR treatment through NF-*κ*B inhibition. Moreover, miR-199a-dependent cross-talk between VEGF and hypoxia-inducible factor 1*α* was validated in the diabetic retina [34], and miR-199a targeting could alleviate DR progression. Given that a single miRNA can regulate multiple genes, and one gene can potentially be regulated by multiple miRNAs, the aberrantly expressed miRNAs investigated in this study could be modulated in different pathways and, thus, might function in the development of DR.

We also found that miR-125b downregulation was correlated with the upregulation of a target gene, with our results suggesting SP1 as a previously unrecognized miR-125b-5p target. Accordingly, in ARPE-19 cells, miR-125b upregulation abolished the high levels of SP1 induced by hyperglycemia. SP1 is a key transcription factor; therefore, aberrant SP1 expression is involved in various types of diseases and plays vital roles in angiogenesis, inflammation, and cell proliferation, migration/invasion, and survival. Interactions between miRNAs and transcription factors are closely related during disease progression, and modulation of this network might exert beneficial effects [35, 36]. Investigation of SP1 at different stages of DR progression revealed that SP1 is tightly associated with angiogenesis-related factors, especially VEGF, and positively regulates their expression [16, 37, 38]. SP1 mRNA is also highly expressed in epiretinal membranes in proliferative DR, with SP1 protein mainly colocalized with VEGF [37]. Furthermore, SP1 binds to the VEGF-A promoter and upregulates VEGF-A expression during DR induced by hyperglycemia [38]. However, additional SP1-related downstream molecules need to be identified in association with DR pathogenesis. Given that modulation of SP1 expression could alleviate DR progression, the application of miR-125b or other miRNAs to target SP1 and other downstream targets might produce beneficial effects in DR treatment.

## 5. Conclusions

This study demonstrated that hyperglycemia induced a decrease in cell viability and an increase in apoptosis of HRECs and RPE cells. The miRNAs identified here showed changes in expression in HRECs and RPE cells along with increases in the duration of hyperglycemia exposure and in the retinas of DM rats along with the development of DR. Furthermore, these miRNAs could exhibit distinct alterations in different layers of the retina or during the different stages of DR. Moreover, SP1 was identified as a potential target of miR-125b-5p associated with DR progression. These results indicated the considerable potential of miRNAs for application in DR-specific therapeutic strategies.
